# Impact of dose-adjusted tacrolimus exposure phenotype on outcomes in kidney transplantation: a large-scale multicenter cohort study

**DOI:** 10.3389/fimmu.2026.1811657

**Published:** 2026-04-15

**Authors:** Donghyun Lim, Sangwan Kim, Ahram Han, Jayeon Ahn, Young Hoon Kim, Kyu Ha Huh, Jae Berm Park, Sun Cheol Park, Jongwon Ha, Sangil Min

**Affiliations:** 1 Department of Surgery, Seoul National University College of Medicine, Seoul, Republic of Korea; 2 Biomedical Research Institute, Seoul National University Hospital, Seoul, Republic of Korea; 3 Department of Kidney and Pancreas Transplantation, Department of Surgery, Asan Medical Center, University of Ulsan College of Medicine, Seoul, Republic of Korea; 4 Department of Surgery, Shinchon Severance Hospital, Yonsei University College of Medicine, Seoul, Republic of Korea; 5 Department of Surgery, Samsung Medical Center, Sungkyunkwan University School of Medicine, Seoul, Republic of Korea; 6 Division of Vascular and Transplant Surgery, Department of Surgery, College of Medicine, The Catholic University of Korea, Seoul, Republic of Korea; 7 Transplantation Center, Seoul National University Hospital, Seoul, Republic of Korea

**Keywords:** dose-adjusted exposure phenotypes, graft survival, immunosuppression, kidney transplantation, pharmacokinetics, tacrolimus

## Abstract

**Introduction:**

Dose-adjusted tacrolimus exposure phenotype, expressed as the trough concentration-to-dose ratio (C0/Dose), may influence post-transplant outcomes, but supporting evidence remains limited.

**Methods:**

We evaluated the association between tacrolimus exposure phenotype and short- and long-term allograft and patient outcomes in a multicenter retrospective cohort study using clinical data warehouse records from five tertiary transplant centers in South Korea (2005–2020). Patients were classified into fast- or slow-dose-adjusted exposure phenotypes using trajectory clustering. Primary outcomes included 1-year endpoints (graft failure, biopsy-proven acute rejection, *de novo* donor-specific antibodies, severe infection, cardiovascular events, malignancy, and death). Secondary outcomes were assessed using the same endpoints for 2–6 years. Inverse probability of treatment weighting (IPTW) and weighted Cox proportional hazards models were used to adjust for confounders.

**Results:**

Among 5,965 recipients, 4,595 (77.0%) were classified as fast-exposure phenotype and 1,370 (23.0%) as slow-exposure phenotypes. Slow-exposure phenotypes received lower tacrolimus doses (time-weighted median 2.1 vs 4.6 mg/day, P < 0.001) but had higher trough levels (6.7 vs 6.1 ng/mL, P < 0.001), higher variability (27.8 vs 26.4%, P < 0.001), and greater time above the therapeutic range (19.7 vs 11.6%, P < 0.001). After IPTW, 1-year efficacy outcomes (biopsy-proven acute rejection, graft failure, and de novo donor-specific antibody development) did not differ significantly between groups. During years 2–6, slow-exposure phenotypes had higher risks of all-cause mortality (adjusted HR 1.616, 95% CI, 1.069–2.442), serious infection requiring hospitalization (adjusted HR 1.537, 95% CI, 1.166–2.026), and malignancy (adjusted HR 1.628, 95% CI, 1.077–2.460).

**Discussion:**

A slow tacrolimus exposure phenotype (high C0/Dose) may serve as a risk marker of cumulative overexposure, associated with increased late mortality, serious infection, and malignancy. Phenotype-informed longitudinal exposure monitoring may improve long-term risk stratification, although whether targeted dose adjustment improves outcomes requires prospective interventional studies.

## 1 Introduction

Kidney transplantation remains the most effective treatment for end-stage kidney disease (ESKD), offering significant improvements in survival rates and quality of life compared with other renal replacement therapies (1, 2). Advances in immunosuppressive therapies have markedly reduced the incidence of acute rejection episodes after transplantation, thereby contributing to improved short-term graft outcomes (3, 4). Currently, the most commonly adopted immunosuppressive regimen after kidney transplantation includes a combination of tacrolimus, mycophenolic acid, and corticosteroids (4, 5). This regimen was designed to prevent graft rejection, while minimizing the risk of adverse effects.

Tacrolimus, a critical component of immunosuppression maintenance, plays a pivotal role in graft survival. Its metabolism is significantly influenced by genetic factors, particularly cytochrome P450 3A5 (CYP3A5) genotype (6). Patients with different CYP3A5 genotypes require substantially different tacrolimus doses to achieve the desired therapeutic drug concentrations. For instance, individuals with the fast dose-adjusted exposure phenotype require approximately 50% more tacrolimus to reach effective blood levels than those with the slow phenotype (7). This variation in metabolism can lead to increased drug exposure and potential adverse effects, including infections, malignancies, nephrotoxicity, neurotoxicity, dyslipidemia, diabetes, and hypertension (8). For this reason, it is of great importance to monitor tacrolimus concentrations continuously in patients undergoing treatment with tacrolimus. Furthermore, studies have been conducted to determine optimal target tacrolimus trough levels (4, 9).

Despite ongoing research, the relationship between dose-adjusted tacrolimus exposure phenotype, therapeutic drug levels, and long-term graft outcomes remains underexplored. Previous studies utilizing data from the Korean Organ Transplantation Registry (KOTRY) have demonstrated associations between tacrolimus metabolism, acute rejection, and delayed graft function (10). However, these studies have been limited by their focus on metabolic rates at only 6 months and 1 year post-transplant, thus missing critical variations in drug dosing and levels over time.

Given the limitations of previous research, there is a pressing need for studies that explore the relationship between individual tacrolimus exposure phenotypes, measured by the tacrolimus trough level (C0) per dose, and short- and long-term graft outcomes. This includes examining outcomes such as biopsy-proven acute rejection (BPAR), graft function, *de novo* donor-specific antibodies (dnDSA), death-censored graft survival, infections, cardiovascular events, malignancies, and overall mortality. To address these gaps, this study proposes a retrospective analysis of clinical data accumulated in Clinical Data Warehouses (CDWs) across multiple institutions in South Korea. CDWs are powerful platforms that facilitate the storage, retrieval, and analysis of extensive clinical data (11), enabling researchers to uncover new patterns and relationships from real-world observational longitudinal data (12). The use of CDW data from major transplant centers in South Korea provides a unique opportunity to derive evidence-based insights into tacrolimus exposure phenotype and its impact on graft outcomes, thereby enhancing the personalization of immunosuppressive therapy and ultimately improving patient care.

## 2 Materials and methods

### 2.1 Study design and data source

This multicenter retrospective cohort study used electronic medical record data from the CDWs of five tertiary transplant centers in South Korea: Seoul National University Hospital, Severance Hospital, Seoul St. Mary’s Hospital, Samsung Medical Center, and Asan Medical Center. This analysis was conducted using the same database infrastructure and patient cohort described in our previous investigation of optimal tacrolimus trough levels, which defined cohort entry on postoperative day 60 (POD 60) to focus on the maintenance immunosuppression period after initial dose stabilization (9). The study was approved by the Institutional Review Board (IRB) of Seoul National University Hospital (IRB No. 2107-196-1237), Asan Medical Center (IRB No. 2022-0139), Severance Hospital (IRB No. 4-2021-1377), and Seoul St. Mary’s Hospital (IRB No. KC21WIDI0910), and the Samsung Medical Center (IRB No. 2022-03-133-002). Given the retrospective nature of this study, the requirement for informed consent was waived.

### 2.2 Study population

Adult patients (≥ 18 years) who underwent kidney transplantation from living or deceased donors between January 1, 2005, and December 31, 2020, were eligible for inclusion. Patients were required to receive tacrolimus as their primary calcineurin inhibitor, with continuous trough level monitoring. Exclusion criteria were age <18 years at transplantation, multi-organ transplantation, re-transplantation, loss to follow-up at the transplantation institution, and fewer than five C0/dose measurements between POD 60 and the end of follow-up.

Patients with comorbidities including diabetes mellitus, hypertension, and prior malignancy were included without restriction. These conditions were incorporated as baseline covariates in the inverse probability of treatment weighting (IPTW) model to minimize confounding.

For trajectory-based clustering analysis, at least five C0/dose measurements were required to ensure reliable phenotype classification. Follow-up for each patient was censored at the occurrence of a primary outcome event (graft failure, BPAR, dnDSA, serious infection, cardiovascular event, malignancy, or death), loss to follow-up, or at the end of the 6-year observation period, whichever occurred first. Consequently, patients who experienced early events before accumulating five measurements on POD 60 were excluded from the analysis.

### 2.3 Tacrolimus exposure variables

The dose-adjusted tacrolimus exposure phenotype (hereafter, exposure phenotype) was defined as the dose-corrected trough concentration (C0/dose) calculated by dividing the morning tacrolimus trough level by the total daily dose administered on the previous day. The C0/dose ratio was calculated by dividing the measured trough level by the drug dose prescribed during the previous outpatient visit. We excluded tacrolimus level measurements > 25 ng/mL to rule out outliers and selected the lowest value when multiple measurements were recorded on the same day.

### 2.4 Trajectory clustering

All C0/dose measurements obtained between POD 60 and the occurrence of any primary outcome or end of follow-up were used to construct individual tacrolimus exposure phenotype trajectories. Trajectory clustering was applied to classify the patients into tacrolimus exposure phenotype groups. Models with two to five clusters were tested, and a two-cluster solution was selected based on clinical interpretability and adequate cluster sizes, yielding fast-dose-adjusted and slow-dose-adjusted exposure phenotype groups.

To address potential reverse causality (i.e., outcome-imminent changes influencing phenotype classification), we evaluated phenotype stability prior to events by reassessing phenotype assignment using serial measurements up to each time point and quantifying any pre-event phenotype switching.

### 2.5 Tacrolimus exposure metrics

Three key exposure metrics were derived for each patient to characterize tacrolimus pharmacokinetics:

Mean tacrolimus dose and trough level: Time-weighted mean values were calculated to account for varying intervals between measurements. For each patient, the contribution of each measurement was weighted by the duration before the next measurement.

Intrapatient variability (IPV): The standard deviation of tacrolimus trough levels across all measurements during follow-up was calculated for each patient. IPV was also expressed as the coefficient of variation (standard deviation divided by mean trough level, multiplied by 100) to normalize for differences in mean exposure.

Time above therapeutic range (TAR): The proportion of follow-up time during which tacrolimus trough levels exceeded the upper limit of the therapeutic range was estimated using the Rosendaal method. This approach employs linear interpolation between consecutive C0 measurements to estimate the percentage of time spent above target levels, thereby providing a continuous measure of cumulative overexposure. For TAR calculations, the target tacrolimus trough range was specified as 5–8 ng/mL and TAR was defined as the proportion of time with interpolated trough concentrations >8 ng/mL.

### 2.6 Outcomes

The primary outcome was a composite clinical endpoint within one year post-transplant, defined as the first occurrence of graft failure (return to dialysis >3 months, re-transplantation, or nephrectomy), BPAR according to the Banff 2013 criteria, dnDSA (MFI >500), severe infection requiring hospitalization, major cardiovascular events (myocardial infarction, ischemic stroke, or coronary intervention), malignancy, or death. Additionally, renal allograft function was assessed longitudinally using estimated glomerular filtration rate (eGFR), calculated from standardized serum creatinine with the 2021 Chronic Kidney Disease Epidemiology Collaboration (CKD-EPI) creatinine equation (13). eGFR was summarized for each patient using the same time-weighted approach applied to tacrolimus exposure metrics to account for unequal intervals between measurements.

Secondary outcomes included the same endpoints assessed 2–6 years post-transplantation among patients who survived beyond 1 year.

### 2.7 Statistical analysis

Baseline characteristics were summarized using means with standard deviations for continuous variables, and frequencies with percentages for categorical variables. Standardized mean differences (SMD) were calculated to assess covariate balance between the groups.

IPTW was applied to balance baseline characteristics between the exposure phenotype groups. The propensity score model for IPTW included baseline covariates only. Time-dependent Cox proportional hazards models incorporating the marginal structural model methodology were then used to estimate hazard ratios, adjusting for baseline covariates and longitudinal tacrolimus dose and trough level information.

The stabilized weights were calculated as the product of the treatment and censoring weights. Tacrolimus levels at 1, 3, 6, and 12 months were adjusted for short-term outcomes. For long-term outcomes, the levels at 1, 2, 3, 4, 5, and 6 years were used. Kaplan-Meier curves were generated, and log-rank tests were performed for survival comparisons. All analyses were conducted using the R software (version 4.4.2; R-project, Institute for Statistics and Mathematics, Vienna, Austria). Statistical significance was set at P < 0.05.

The propensity score model for IPTW included all baseline covariates listed in **Table 1**: recipient age, sex, body mass index (BMI), blood type, hypertension, diabetes mellitus, primary etiology of ESKD, dialysis type and duration, calculated panel reactive antibody (cPRA; Class I and II), donor specific antibody (DSA) positivity, number of human leukocyte antigen (HLA) mismatches, desensitization status, donor age, sex, BMI, donor type (living vs. deceased). Cold ischemia time was initially considered but excluded from the propensity score model because of substantial missing data in living donor transplants. Although cold ischemia time is reported in **Table 1**, it showed residual imbalance after weighting (SMD 0.199), inherently reflecting its correlation with donor type. Standardized mean differences for each covariate were evaluated using a love plot to assess covariate balance before and after weighting. Stabilized IPTW weights were truncated at the 1st and 99th percentiles to prevent undue influence of extreme weights.

**Table 1:** Baseline characteristics on the transplant date by exposure phenotype group.

Variable	Tacrolimus exposure phenotype (C0/Dose)		P-value	SMD
	Fast	Slow		
Recipient	4,595	1,370		
Age, y	45.9 ± 11.8	51.1 ± 11.5	<0.001	0.442
Sex (Female), n(%)	2095 (45.6%)	447 (32.6%)	<0.001	0.268
Body mass index(BMI), kg/m^2^	22.8 ± 5.2	23.6 ± 7.8	0.287	0.114
Blood type, n(%)			0.404	0.118
RH+	2635 (57.3%)	915 (66.8%)		
A	912 (19.8%)	319 (23.3%)		
B	705 (15.3%)	267 (19.5%)		
AB	334 ( 7.3%)	117 ( 8.5%)		
RH-	6 ( 0.2%)	3 ( 0.2%)		
A	2 ( 0.0%)	2 ( 0.1%)		
B	1 ( 0.0%)	1 ( 0.1%)		
AB	1 ( 0.0%)	0 ( 0.0%)		
* N/A	1,954 (42.5%)	452 (33.0%)		
Hypertension, n(%)	3205 (69.7%)	1001 (73.1%)	0.02	0.129
Diabetes mellitus, n(%)	905 (19.7%)	365 (26.6%)	<0.001	0.146
Primary etiology of ESRD, n(%)			<0.001	0.212
DM	835 (18.2%)	340 (24.8%)		
HTN	512 (11.1%)	160 (11.7%)		
GN	797 (17.3%)	221 (16.1%)		
PKD	243 ( 5.3%)	92 ( 6.7%)		
IgA	605 (13.2%)	138 (10.1%)		
others	563 (12.3%)	117 ( 8.6%)		
unknown	1040 (22.6%)	302 (22.0%)		
Dialysis, n(%)	3699 (80.5%)	1155 (84.3%)	0.001	0.107
Type of dialysis, n(%)			<0.001	0.148
Pre-emptive	896 (19.5%)	215 (15.7%)		
Hemodialysis	3019 (65.7%)	913 (66.7%)		
Peritoneal dialysis	501 (10.9%)	164 (12.0%)		
Hemofiltration (Mix)	179 ( 3.9%)	78 ( 5.7%)		
Duration of dialysis, month	36.6 ± 53.6	43.1 ± 54.7	<0.001	0.120
***Transplant information***				
Calculated panel reactive antibody (cPRA)				
Class I	13.0 ± 26.1	9.1 ± 21.4	<0.001	0.166
Class II	11.36 ± 24.5	9.1 ± 21.4	<0.001	0.098
DSA positivity			0.064	0.110
Class I	239 ( 5.2%)	52 ( 3.8%)		
Class II	187 ( 4.1%)	59 ( 4.3%)		
Class I & Class II	76 ( 1.7%)	11 ( 0.8%)		
DSA value of MFI	535.7 ± 1888.7	419.1 ± 1423.7	0.118	0.070
Class I	3517.3 ± 3855.1	2605.3 ± 2595.3	0.073	0.278
Class II	3426.5 ± 4264.5	2698.6 ± 2834.5	0.178	0.201
Number of mismatched of HLA	3.3 ± 1.6	3.2 ± 1.6	0.506	0.021
Desensitization, n(%)	1183 (25.7%)	278 (20.3%)	<0.001	0.130
Number of IVIG	0.8 ± 2.3	0.5 ± 1.9	0.004	0.104
Number of plasmapheresis	1.0 ± 2.3	0.8 ± 2.3	0.009	0.116
Donor				
Age, y	44.9 ± 12.7	45.0 ± 13.3	0.66	0.013
Sex (Female), n(%)	2240 (48.7%)	671 (49.0%)	0.906	0.005
Body mass index(BMI), kg/m^2^	23.9 ± 3.4	23.8± 3.4	0.228	0.040
Blood type, n(%)			0.666	0.068
RH+	2793 (60.7%)	973 (71.0%)		
A	949 (20.7%)	310 (22.6%)		
B	712 (15.5%)	270 (19.7%)		
AB	263 ( 5.7%)	97 ( 7.1%)		
RH-	3 ( 0.1%)	1 ( 0.1%)		
A	2 ( 0.1%)	1 ( 0.1%)		
B	–	–		
AB	–	–		
* N/A	1799 (39.2%)	396 (28.9%)		
Hypertension, n(%)	347 ( 7.6%)	114 ( 8.3%)	<0.001	0.160
Donor type, n(%)			<0.001	0.200
Living	3516 (76.5%)	943 (68.8%)		
Deceased	919 (20.0%)	384 (28.1%)		
* N/A	160 ( 3.5%)	43 ( 3.1%)		
Cold ischemic time (min)	136.3 ± 136.6	154.7 ± 135.6	0.023	0.135

* N/A : not available

## 3 Results

### 3.1 Study population

**Figure 1:**
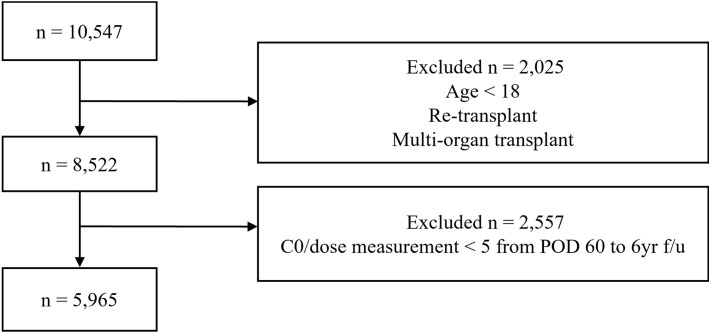
Patient selection flowchart.

Between January 2005 and December 2020, 10,547 kidney transplant recipients from five participating centers were screened (**Figure 1**). A total of 2,025 patients were excluded because of age <18 years, re-transplantation, or multiorgan transplantation. An additional 2,557 patients were excluded due to insufficient C0/dose measurements (<5 values from POD 60 through the end of follow-up or outcome event occurrence), including those who experienced an outcome event after POD 60 but before performing five measurements, as well as those with incomplete monitoring data. The final cohort comprised 5,965 patients.

**Figure 2:**
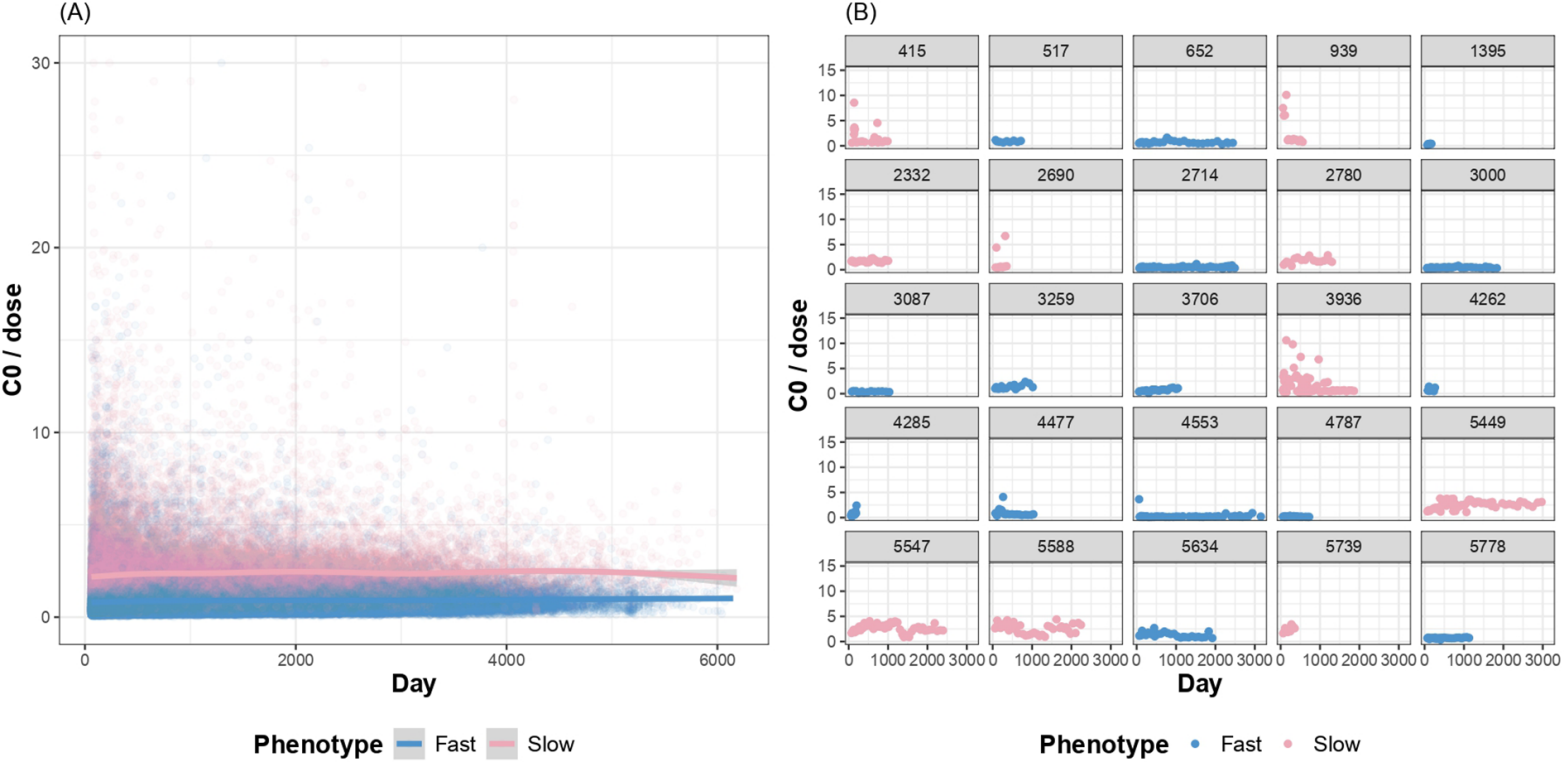
Tacrolimus exposure phenotype trajectory clustering. **(A)** Mean C0/dose trajectories for fast dose-adjusted exposure phenotype (n=4,595) and slow dose-adjusted exposure phenotype (n=1,370). Shaded areas represent 95% confidence intervals. **(B)** Individual trajectories of randomly selected patients, demonstrating stable separation between exposure phenotypes.

Based on the trajectory clustering of longitudinal C0/dose values, 4,595 patients (77.0%) were classified as having a fast-exposure phenotype and 1,370 (23.0%) as a slow-exposure phenotype (**Figure 2**). The two groups demonstrated distinct and stable C0/dose trajectories throughout thefollow-up period. Among the participants who experienced outcomes, phenotype assignment remained stable during the pre-event period with no evidence of phenotype switching (**Supplementary Figure 1**).

### 3.2 Baseline characteristics

Prior to weighting, slow-exposure phenotype recipients were significantly older (mean age 51.1 vs. 45.9 years, P < 0.001) and more likely to be male (67.4% vs. 54.4%, P < 0.001) compared with fast exposure phenotype (**Table 1**). The slow-exposure phenotype group had a higher prevalence of diabetes mellitus (26.7% vs. 19.7%, P < 0.001) and hypertension (73.1% vs. 69.8%, P = 0.02). Additionally, the slow-exposure phenotype was more likely to have received deceased donor transplants (28.1% vs. 20.0%, P < 0.001).

After IPTW adjustment, baseline covariates were well balanced between groups, with standardized mean differences reduced to <0.1 for most variables (**Table 2**). The weighted cohorts were comparable in terms of age, sex, comorbidities, transplant characteristics and donor factors.

**Table 2:** After IPTW, characteristics on the transplant date by exposure phenotype group.

Variable	Tacrolimus exposure phenotype (C0/Dose)		P-value	SMD
	Fast	Slow		
Recipient	5,968.36*	5,987.45*		
Age, y	47.1 ± 11.8	47.1 ± 12.3	0.952	0.003
Sex (Female), n(%)	2531.4 (42.4%)	2491.3 (41.6%)	0.669	0.016
Body mass index(BMI), kg/m^2^	23.0 ± 5.6	22.9 ± 5.7	0.789	0.009
Blood type, n(%)			0.843	0.066
RH+	3554.4 (59.6%)	3570.1 (59.6%)		
A	1230.8 (20.6%)	1233.6 (20.6%)		
B	981.4 (16.4%)	1034.2 (17.3%)		
AB	453.4 ( 7.6%)	469.9 ( 7.8%)		
RH-	7.8 ( 0.1%)	6 ( 0.1%)		
A	2.5 ( 0.0%)	3.1 ( 0.1%)		
B	1.5 ( 0.0%)	2.9 ( 0.0%)		
AB	1.1 ( 0.0%)	0.0 ( 0.0%)		
# N/A	2406.1 (40.3%)	2411.4 (40.3%)		
Hypertension, n(%)	4212.9 (70.6%)	4226.6 (70.6%)	0.996	<0.001
Diabetes mellitus, n(%)	1293.7 (21.7%)	1335.9 (22.3%)	0.65	0.015
Primary etiology of ESRD, n(%)			0.999	0.024
DM	1192.2 (20.0%)	1208.0 (20.2%)		
HTN	677.2 (11.3%)	707.4 (11.8%)		
GN	1007.1 (16.9%)	976.7 (16.3%)		
PKD	330.5 ( 5.5%)	327.1 ( 5.5%)		
IgA	745.0 (12.5%)	723.3 (12.1%)		
others	675.7 (11.3%)	679.3 (11.3%)		
unknown	1340.6 (22.5%)	1365.6 (22.8%)		
Dialysis, n(%)	4857.6 (81.4%)	4916.7 (82.1%)	0.622	0.019
Type of dialysis, n(%)			0.922	0.024
Pre-emptive	1110.7 (18.6%)	1070.7 (17.9%)		
Hemodialysis	3939.2 (66.0%)	4020.0 (67.1%)		
Peritoneal dialysis	660.1 (11.1%)	645.8 (10.8%)		
Hemofiltration (Mix)	258.3 ( 4.3%)	250.8 ( 4.2%)		
Duration of dialysis, month	37.6 ± 53.7	39.0 ± 52.9	0.475	0.026
***Transplant information***				
Calculated panel reactive antibody (cPRA)				
Class I	12.4 ± 25.5	10.0 ± 21.9	0.006	0.103
Class II	10.7 ± 23.8	10.6 ± 22.6	0.888	0.006
DSA positivity			0.190	0.097
Class I	290.9 ( 4.9%)	265.5 ( 4.4%)		
Class II	231.2 ( 3.9%)	272.7 ( 4.6%)		
Class I & Class II	91.8 ( 1.5%)	55.8 ( 0.9%)		
DSA value of MFI	514.6 ± 1835.5	439.7 ± 1506.6	0.307	0.045
Class I	3458.1 ± 3796.2	2614.4 ± 2496.7	0.031	0.263
Class II	3358.8 ± 4175.5	3219.7 ± 3168.1	0.796	0.038
Number of mismatched of HLA	3.2 ± 1.6	3.2 ± 1.6	0.928	0.003
Desensitization, n(%)	1457.1 24.4%)	1395.3 (23.3%)	0.494	0.026
Number of IVIG	0.8 ± 2.2	0.6 ± 2.0	0.063	0.086
Number of plasmapheresis	0.9 ± 2.2	0.9 ± 2.4	0.782	0.011
Donor				
Age, y	44.9 ± 12.8	45.1 ± 12.7	0.673	0.015
Sex (Female), n(%)	2910.9 (48.8%)	2837.0 (47.4%)	0.45	0.028
Body mass index(BMI), kg/m^2^	23.9± 3.3	23.9 ± 3.3	0.955	0.002
Blood type, n(%)			0.753	0.066
RH+	3769.2 (63.1%)	3787.0 (63.2%)		
A	1266.0 (21.2%)	1236.5 (20.7%)		
B	984.3 (16.5%)	1060.3 (17.7%)		
AB	363.0 ( 6.1%)	398.5 ( 6.7%)		
RH-	4.5 ( 0.1%)	4.8 ( 0.1%)		
A	3.4 ( 0.1%)	4.8 ( 0.1%)		
B	–	–		
AB	–	–		
# N/A	2194.6 (36.8%)	2195.7 (36.7%)		
Hypertension, n(%)	462.6 (11.4%)	515.3 (12.3%)	0.527	0.029
Donor type, n(%)			0.641	0.048
Living	4458.7 (74.7%)	4416.9 (73.8%)		
Deceased	1305.8 (21.9%)	1312.8 (22.0%)		
# N/A	196.9 ( 3.4%)	250.9 ( 4.2%)		
Cold ischemic time (min)	134.8 ± 134.0	161.7 ± 137.2	0.005	0.199

* This value represents the weighted pseudo sample size.

# N/A : not available

### 3.3 Tacrolimus exposure patterns

Tacrolimus exposure metrics differed substantially between the exposure phenotype groups (**Table 3**). Slow exposure phenotype required significantly lower daily doses to achieve therapeutic levels: the time-weighted median dose was 2.1 mg/day (interquartile range [IQR] 1.6–2.9) in slow exposure phenotype compared with 4.6 mg/day (IQR 3.2–6.3) in fast exposure phenotype (P < 0.001). Despite these lower doses, slow exposure phenotype maintained higher mean trough concentrations (6.7 ng/mL [IQR 5.7–7.6] vs. 6.1 ng/mL [IQR 5.1–7.0], P < 0.001).

**Table 3:** Tacrolimus exposure metrics (dose, trough level, IPV, and time above therapeutic range) according to metabolism group.

Variable	Tacrolimus exposure phenotype (C0/Dose)		P-value
	Fast	Slow	
Recipient	4,595	1,370	
Drug Dose			
Mean*	4.6 [3.2; 6.3]	2.1 [1.6; 2.9]	<0.001
Trough level (C0)			
Mean*	6.1 [5.1; 7.0]	6.7 [5.7; 7.6]	<0.001
Standard deviation (SD)*	1.5 [1.2; 2.0]	1.8 [1.4; 2.4]	<0.001
Intra patient variability (IPV)*	26.4 [20.6; 34.3]	27.8 [21.7; 36.5]	<0.001
Therapeutic range (Time above range, TAR)	11.6 [3.1; 25.5]	19.7 [9.1; 37.0]	<0.001

* The parameters were produced through the implementation of a time-weighted approach.

IPV, measured as the standard deviation of trough levels, was greater in slow exposure phenotype (1.8 ng/mL [IQR 1.4–2.4] vs. 1.5 ng/mL [IQR 1.2–2.0], P < 0.001). When expressed as the coefficient of variation, the slow-exposure phenotype also demonstrated higher variability (27.8% [IQR 21.7–36.5] vs. 26.4% [IQR 20.6–34.3], P < 0.001). Furthermore, the slow exposure phenotype spent a significantly greater proportion of follow-up time above the therapeutic range: the median TAR was 19.7% (IQR 9.1–37.0) in the slow exposure phenotype versus 11.6% (IQR 3.1–25.5) in the fast exposure phenotype (P < 0.001). These findings indicate that despite lower doses, the slow-exposure phenotype experienced chronic overexposure to tacrolimus.

### 3.4 Primary outcomes (1-year)

During the first year post-transplantation, no significant differences were observed between the exposure phenotype groups for any efficacy or safety outcome components after adjustment (**Table 4**). For efficacy outcomes, crude hazard ratios for graft failure, BPAR, and dnDSA development were all approximately 1.0, without statistical significance, indicating that both fast- and slow-exposure phenotypes achieved comparable immunologic protection during this early period, despite marked differences in tacrolimus dosing requirements.

**Table 4:** Hazard ratio during 1 year post-transplant.

1 Year outcome	Phenotype	N	Outcome	Follow up time (Days)	Incidence	Crude HR	P-value	Adjusted HR	P-value
Death	Fast	4,595	15	364.52	3.27	2.018 (0.833 - 4.612)	0.096	1.405 (0.582 - 3.930)	0.450
	Slow	1,370	9	363.84	6.59				
BPAR	Fast	4,595	177	359.64	39.12	1.098 (0.817 - 1.478)	0.535	1.086 (0.751 - 1.570)	0.662
	Slow	1,370	58	359.77	42.98				
GF	Fast	4,595	27	364.15	5.89	0.996 (0.452 - 2.191)	0.991	0.936 (0.417 - 2.099)	0.871
	Slow	1,370	8	363.74	5.86				
dnDSA	Fast	4,595	23	364.37	5.02	1.316 (0.609 - 2.843)	0.485	0.921 (0.405 - 2.091)	0.843
	Slow	1,370	9	363.87	6.59				
Serious infection	Fast	4,595	133	361.30	29.26	1.116 (0.794 - 1.570)	0.527	1.347 (0.899 - 2.020)	0.149
	Slow	1,370	44	359.87	32.60				
CVE	Fast	4,578	34	362.48	7.48	2.489 (1.485 - 4.171)	<0.001	1.822 (0.867 - 3.829)	0.113
	Slow	1,360	25	358.68	18.72				
Malignancy	Fast	4,568	24	363.38	5.28	1.958 (1.013 - 3.786)	0.048	2.203 (0.981 - 4.945)	0.056
	Slow	1,364	14	362.40	10.34				

**Figure 3:**
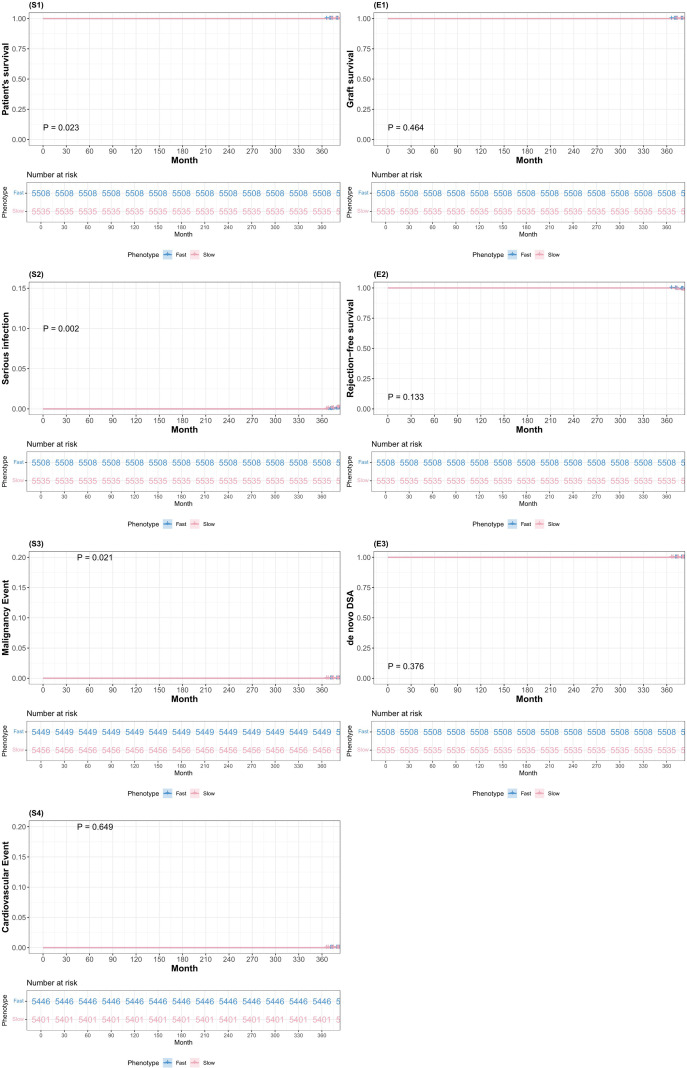
IPTW-adjusted Kaplan-Meier estimates of the 1-year composite clinical endpoint comparing fast vs slow tacrolimus C0/dose trajectory-derived exposure phenotypes. Panels S1–S4 (left column) depict safety endpoints: patient survival (S1), serious infection requiring hospitalization (S2), malignancy (S3), and cardiovascular events (S4). Panels E1–E3 (right column) depict efficacy endpoints: graft survival free from failure (E1), rejection-free survival (biopsy-proven acute rejection) (E2), and *de novo* donor-specific antibody (dnDSA) development (E3). For each endpoint, inverse probability of treatment weighting (IPTW)-adjusted Kaplan–Meier curves are shown separately for fast and slow exposure phenotypes, with the number at risk displayed below each plot and adjusted log-rank P values provided within panels.

Among the safety outcomes, the death and severe infection rates were similar between the groups. However, crude hazard ratios suggested potentially higher risks of cardiovascular events (cHR 2.49, 95% CI 1.49–4.17, P < 0.001) and malignancy (cHR 1.96, 95% CI 1.01–3.79, P = 0.048) in the slow-exposure phenotype (**Supplementary Figure 2**). These associations were attenuated and became non-significant after IPTW adjustment (aHR 1.82, P = 0.113 and aHR 2.20, P = 0.056, respectively), suggesting that baseline differences in patient characteristics, such as older age and higher comorbidity burden in the slow-exposure phenotype, accounted for most of the observed crude differences. Consistent with the weighted Cox models, the IPTW-adjusted Kaplan–Meier curves showed largely overlapping event-free survival for the 1-year composite clinical endpoint between the fast- and slow-exposure phenotype groups (**Figure 3**). Nevertheless, the borderline significance of the adjusted malignancy risk (P = 0.056) suggested an emerging safety signal that would become more apparent during longer follow-up periods.

### 3.5 Secondary outcomes (2–6 years)

Among 5,505 patients followed beyond 1 year (4,250 fast-exposure and 1,255 slow-exposure phenotypes), significant differences emerged in safety outcomes, while efficacy remained comparable (**Table 5**).

**Table 5:** Hazard ratio during 2–6 year post-transplant.

2–6 Year outcome	Phenotype	N	Outcome	Follow up time (Days)	Incidence	Crude HR	P-value	Adjusted HR	P-value
Death	Fast	4,250	66	1,786.51	3.17	2.257 (1.537 - 3.315)	<0.001	1.616 (1.069 - 2.442)	0.023
	Slow	1,255	43	1,754.21	7.13				
BPAR	Fast	4,250	403	1,689.26	20.50	1.456 (1.214 - 1.745)	<0.001	1.185 (0.950 - 1.478)	0.133
	Slow	1,255	165	1,612.75	29.78				
GF	Fast	4,250	147	1,761.48	7.17	1.076 (0.773 - 1.499)	0.664	1.164 (0.775 - 1.747)	0.464
	Slow	1,255	46	1,741.28	7.69				
dnDSA	Fast	4,250	224	1,675.33	11.49	1.125 (0.865 - 1.463)	0.381	1.140 (0.853 - 1.522)	0.376
	Slow	1,255	74	1,668.17	12.91				
Serious infection	Fast	4,250	291	1,655.98	15.10	1.170 (0.932 - 1.469)	0.175	1.537 (1.166 - 2.026)	0.002
	Slow	1,255	100	1,648.88	17.65				
CVE	Fast	4,207	42	1,714.55	2.13	2.160 (1.325 - 3.523)	0.002	1.149 (0.632 - 2.087)	0.649
	Slow	1,222	26	1,696.03	4.58				
Malignancy	Fast	4,205	81	1,705.67	4.12	2.308 (1.636 - 3.257)	<0.001	1.628 (1.077 - 2.460)	0.021
	Slow	1,241	54	1,679.77	9.46				

**Figure 4:**
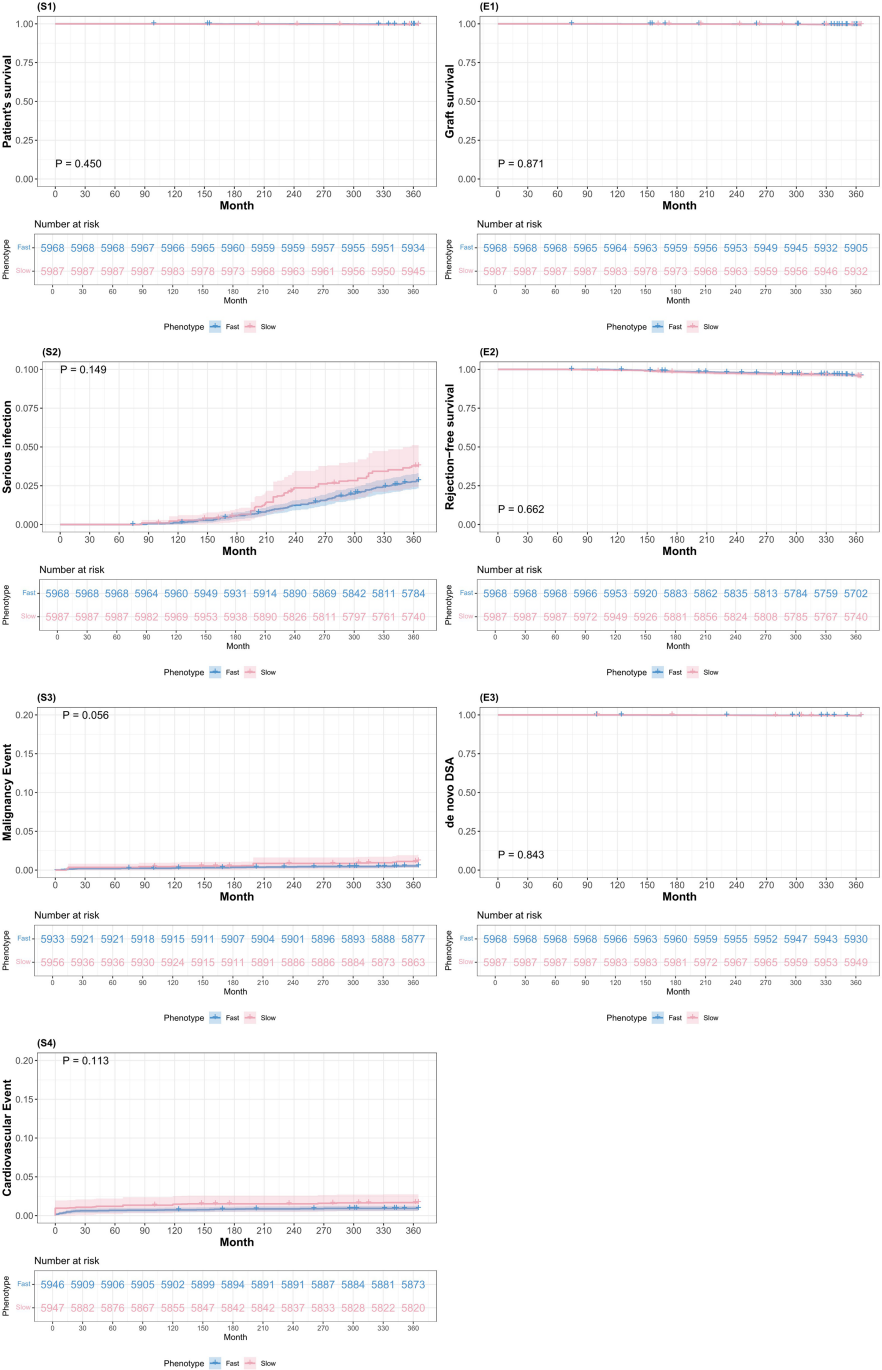
IPTW-adjusted Kaplan-Meier estimates of the 2–6 year composite clinical endpoint comparing fast vs slow tacrolimus C0/dose trajectory-derived exposure phenotypes. Panels S1–S4 (left column) depict safety endpoints: patient survival (S1), serious infection requiring hospitalization (S2), malignancy (S3), and cardiovascular events (S4). Panels E1–E3 (right column) depict efficacy endpoints: graft survival free from failure (E1), rejection-free survival (biopsy-proven acute rejection) (E2), and *de novo* donor-specific antibody (dnDSA) development (E3). For each endpoint, inverse probability of treatment weighting (IPTW)-adjusted Kaplan–Meier curves are shown separately for fast and slow exposure phenotypes, with the number at risk displayed below each plot and adjusted log-rank P values provided within panels.

Slow exposure phenotype had significantly higher risks of all-cause mortality (aHR 1.62, 95% CI 1.07–2.44, P = 0.023), severe infection requiring hospitalization (aHR 1.54, 95% CI 1.17–2.03, P = 0.002), and malignancy (aHR 1.63, 95% CI 1.08–2.46, P = 0.021) (**Figure 4**). These findings indicate that the slow-exposure phenotype confers a 54–63% increased risk of these major safety endpoints during the maintenance phase, even after adjustment for baseline confounders. The consistency of the effect sizes across mortality, infection, and malignancy (all hovering around 1.5 to 1.6) suggests a common underlying mechanism likely related to cumulative tacrolimus overexposure, as evidenced by the higher mean trough levels, greater IPV, and prolonged TAR observed in this group.

In contrast, the efficacy outcomes during years 2–6 remained comparable between groups. Graft failure (aHR 1.16, 95% CI 0.78–1.75, P = 0.464), BPAR (aHR 1.19, 95% CI 0.95–1.48, P = 0.133), and dnDSA development (aHR 1.14, 95% CI 0.85–1.52, P = 0.376) showed no significant differences, confirming that the lower tacrolimus doses administered to slow exposure phenotype provided adequate immunosuppression. Cardiovascular events, which were elevated in the crude analysis (**Supplementary Figure 3**), were not significantly different after adjustment (aHR 1.15, P = 0.649), possibly reflecting the successful management of modifiable cardiovascular risk factors in this high-risk population. Among recipients followed beyond 1 year, IPTW-adjusted Kaplan–Meier estimates demonstrated lower event-free survival for the composite clinical endpoint in the slow-exposure phenotype during years 2–6.

The time-weighted mean eGFR did not differ significantly between the fast- and slow-exposure phenotype groups (P = 0.315), despite visually distinct but largely overlapping longitudinal trajectories (**Supplementary Figure 4**).

## 4 Discussion

The dose-adjusted tacrolimus exposure phenotype, defined by longitudinal C0/dose trajectories, showed a clearer association with long-term patient safety than classical efficacy endpoints in this cohort. Although the slow-exposure phenotype received lower tacrolimus doses, it exhibited higher trough concentrations, greater IPV, and a longer TAR. This pharmacokinetic profile indicated that the slow-exposure phenotype experienced chronic or intermittent overexposure, despite achieving acceptable trough targets. A critical insight is that conventional trough-based monitoring, although adequate for preventing rejection, does not capture the cumulative exposure burden, which may be relevant to long-term toxicity. Despite these exposure differences, graft failure, BPAR, and dnDSA development were not significantly different after adjustment, whereas death, severe infection, and malignancy were consistently higher among the slow-exposure phenotypes over longer follow-up periods.

Our results complement the existing literature, which has traditionally focused on the fast-exposure phenotype as the primary high-risk group requiring intensified monitoring. Previous studies have largely emphasized the risk profile of the fast-exposure phenotype (low C0/dose), reporting associations with inferior renal function and, in some settings, higher rejection-related events. Studies using single time-point C0/dose measurements have linked low C0/dose to worse graft-related outcomes and have suggested potential pharmacogenetic contributions (7, 10, 14, 15). More recent large-scale analyses have also evaluated the prognostic impact of a low C0/dose at 1 year on graft loss and mortality, highlighting that metabolic surrogates may capture clinically relevant vulnerability beyond a single trough value (16). In contrast, our trajectory-based classification focuses on sustained metabolism patterns from postoperative day 60 onward and may, therefore, be more sensitive to chronic overexposure patterns in the slow-exposure phenotype, particularly when combined with variability and time above the range. These seemingly contradictory findings may reflect different outcome domains; while fast exposure phenotypes appear vulnerable to graft-related complications driven by underexposure or high peak concentrations, slow exposure phenotypes may accumulate risk for systemic toxicities, including infection and malignancy, through higher cumulative exposure burden over time. This dual risk profile is consistent with findings from a Korean single-center study that demonstrated that early tacrolimus underexposure (<7 ng/mL within the first month) was independently associated with poor graft survival, whereas early overexposure (>10 ng/mL within two months) increased the risk of infection and post-transplant diabetes mellitus (17). This interpretation is further supported by observational data demonstrating that a higher tacrolimus exposure intensity during the first year is associated with an increased long-term cancer risk in kidney transplant recipients (18). Taken together, both extremes of the dose-adjusted exposure spectrum may confer risks, albeit through distinct mechanisms.

These findings should not be interpreted as negating the well-established graft-related risks associated with fast-exposure phenotypes. Rather, they highlight a complementary long-term safety concern in the opposite direction—that slow-exposure phenotypes may accumulate systemic toxicity risks through chronic overexposure, despite adequate immunologic protection. The apparent divergence from prior C0/D literature likely reflects methodological differences; whereas classical studies assign metabolizer status based on a single measurement at a fixed time point (e.g., 3 or 12 months post-transplant), our trajectory-based classification captures sustained metabolic patterns from POD 60 onward, making it inherently more sensitive to chronic overexposure patterns in the slow-exposure phenotype rather than acute underexposure events.

The observed excess of severe infection and malignancy in the slow-exposure phenotype is biologically plausible in the context of cumulative or intermittent immunosuppression. A systematic review and meta-analysis comparing standard versus reduced tacrolimus exposure suggested that higher exposure reduces acute rejection without clear differences in graft loss or patient survival, underscoring that “more tacrolimus” does not necessarily translate into better long-term outcomes (19). In addition, tacrolimus exposure intensity has been associated with post-transplant cancer risk in observational data, supporting the concept that sustained higher tacrolimus exposure may contribute to oncogenic risk over time. Meta-analytical evidence also suggests an overall association between tacrolimus use and malignancy risk, reinforcing the need to balance immunosuppressive efficacy with late complications (20). Taken together, our findings support a safety-oriented interpretation that dose-adjusted exposure phenotypes may matter the most when they push patients toward recurrent overexposure rather than underexposure.

The association between the slow-exposure phenotype and increased malignancy risk should be interpreted with caution. Regarding cancer screening, all Korean citizens are eligible for the National Cancer Screening Program (NCSP), which provides standardized screening for major cancers (gastric, colorectal, liver, breast, and cervical cancer) at regular intervals, typically every two years. This population-level screening program reduces the likelihood of substantial differential ascertainment bias between exposure phenotype groups. Nevertheless, transplant-specific enhanced surveillance protocols may vary across centers, and individual adherence to screening was not captured. Therefore, the dose-adjusted exposure phenotype may function as a surrogate marker of overall immunosuppression burden rather than a direct causal driver of malignancy.

Although the absolute difference in mean trough concentrations between exposure phenotypes was modest (6.7 vs. 6.1 ng/mL), the TAR difference is substantial (19.7% vs. 11.6%, a 70% relative increase in time above therapeutic range), reflecting meaningfully different overexposure burdens. These time-weighted metrics, when accumulated over years of follow-up, translate into substantially different cumulative exposure when integrated over time. With respect to center-specific treatment strategies, we recognize that some degree of center effect is likely to exist. However, we conducted the analyses under the assumption that all participating centers—each with high graft and patient survival rates—apply broadly comparable, standard-of-care immunosuppressive and clinical management practices. Detailed longitudinal steroid dosing data and mTOR inhibitor use were not systematically captured across all five CDWs and represent a priority for future multi-center prospective studies.

Clinically, these data suggest that the dose-adjusted exposure phenotype can be used to guide more individualized monitoring strategies. For patients classified as having a slow-exposure phenotype, clinicians may consider tighter surveillance of exposure metrics beyond a single trough value, such as variability and time above the range and earlier intervention when persistent overexposure is suspected (21). Evidence from cohorts and reviews has increasingly emphasized that C0/dose and related exposure measures may complement traditional therapeutic drug monitoring by identifying patients who remain at risk despite “acceptable” trough targets (7, 14, 15). Our previous investigations demonstrated that high IPV is associated with early deterioration of chronic histological lesions and calcineurin inhibitor nephrotoxicity in kidney transplant recipients, further reinforcing the clinical relevance of monitoring exposure variability beyond mean trough levels (22, 23).

Notably, despite the evidence of cumulative overexposure in the slow-exposure phenotype, graft failure did not differ significantly between groups. This may reflect the possibility that the overexposure burden manifests primarily as systemic immunosuppression-related harm rather than direct nephrotoxicity. Indeed, a supplementary analysis showed no significant between-group difference in time-weighted mean eGFR (P = 0.315), and the longitudinal eGFR curves were largely overlapping throughout follow-up (**Supplementary Figure 4**), supporting this interpretation. Additionally, the relatively short follow-up for detecting chronic nephrotoxicity and competing risks from mortality and infection may have further attenuated the graft failure signal.

This study has some limitations. First, requiring C0/dose measurements from postoperative day 60 onward and at least five available values excluded patients with early instability, early graft loss, or early death, potentially biasing the cohort toward more stable survivors and limiting inference for the immediate post-transplant period. This design may also have preferentially excluded high-risk events related to the fast-exposure phenotype occurring early after transplantation, thereby attenuating risk signals in that group. Future studies using formal landmark analysis with time-varying phenotype assignment or prospective designs that capture early instability are warranted. Second, IPV was assessed using between-visit trough fluctuations rather than within-day pharmacokinetic sampling. Because variability was expressed as the standard deviation around the mean, groups with higher mean tacrolimus levels may appear to have lower normalized variability despite clinically meaningful absolute fluctuations. Third, the retrospective design and reliance on routine clinical data introduced the possibility of residual confounding and exposure misclassification. Important factors such as medication adherence, lifestyle, concomitant medications affecting tacrolimus pharmacokinetics, viral infection status, smoking history, and cumulative steroid exposure were not systematically captured and may represent sources of residual confounding. Although the Korean National Cancer Screening Program provides standardized population-level cancer screening every two years, transplant-specific enhanced surveillance protocols and individual adherence were not available in the CDW data, limiting the interpretation of the malignancy findings. Detailed comorbidity-specific subgroup analyses were not performed due to sample size constraints for individual conditions. Fourth, although a supplementary analysis of longitudinal eGFR trajectories showed no significant between-group difference, protocol biopsy-based chronic lesion assessment (including interstitial fibrosis/tubular atrophy and calcineurin inhibitor nephrotoxicity scores) was not available across all centers, precluding direct histologic evaluation of the relationship between tacrolimus exposure phenotype and chronic graft injury. Therefore, while the consistency of the safety signal across multiple endpoints is reassuring, these findings should be interpreted as hypothesis-generating, and prospective metabolism-guided interventional studies are needed to confirm causality and refine exposure targets.

In conclusion, dose-adjusted tacrolimus exposure phenotype was associated with distinct risk profiles in kidney transplant recipients. The slow dose-adjusted exposure phenotype showed comparable efficacy outcomes but was associated with higher long-term risks of death, severe infection, and malignancy. These findings suggest that sustained or recurrent tacrolimus overexposure, despite acceptable trough levels, may represent an under-recognized source of late adverse outcomes. Incorporating the exposure phenotype and dynamics into routine therapeutic drug monitoring may enable improved long-term risk stratification and support more individualized, safety-oriented tacrolimus management beyond the early post-transplant period. Patients exhibiting a high-risk profile of the slow-exposure phenotype, combined with high variability and prolonged supratherapeutic exposure, may warrant intensified safety surveillance; however, whether targeted dose minimization in this population improves outcomes remains to be determined through prospective interventional studies. Future studies focusing on tacrolimus exposure phenotype-guided dosing strategies are warranted to validate these findings and refine the outcome-driven immunosuppression protocols.

## Data Availability

The datasets presented in this article are not readily available because Data supporting the findings of this study are available from the corresponding author upon reasonable request. However, owing to the nature of the data and policies of the institutional review board (IRB), some restrictions may apply to the availability of these data. Requests to access the datasets should be directed to surgeonmsi@gmail.com.

## Supplementary material

Supplementary Figure 1 Pre-event tacrolimus C0/dose trajectories among kidney transplant recipients with the longest follow-up. Serial tacrolimus C0/dose measurements from postoperative day 60 until just before the first occurrence of a clinical outcome or censoring are displayed for the 25 patients with the longest follow-up. Each line represents an individual trajectory, color-coded by outcome type: death (red), graft failure (yellow), biopsy-proven acute rejection (orange), *de novo* donor-specific antibody development (green), and severe infection requiring hospitalization (purple). Trajectories are overlaid on the fast and slow dose-adjusted exposure phenotype bands, and all patients remain within their originally assigned exposure phenotype without crossing between bands during the pre-event period, indicating no evidence of phenotype switching prior to outcome occurrence.

Supplementary Figure 2 Crude Kaplan-Meier estimates of the 1-year composite clinical endpoint comparing fast vs slow tacrolimus C0/dose trajectory-derived exposure phenotypes. Panels S1–S4 (left column) depict safety endpoints: patient survival (S1), serious infection requiring hospitalization (S2), malignancy (S3), and cardiovascular events (S4). Panels E1–E3 (right column) depict efficacy endpoints: graft survival free from failure (E1), rejection-free survival (biopsy-proven acute rejection) (E2), and *de novo* donor-specific antibody (dnDSA) development (E3). For each endpoint, unadjusted Kaplan–Meier curves are shown separately for fast and slow exposure phenotypes, with the number at risk displayed below each plot and log-rank P values provided within panels.

Supplementary Figure 3 Crude Kaplan-Meier estimates of the 2–6 year composite clinical endpoint comparing fast vs slow tacrolimus C0/dose trajectory-derived exposure phenotypes. Panels S1–S4 (left column) depict safety endpoints: patient survival (S1), serious infection requiring hospitalization (S2), malignancy (S3), and cardiovascular events (S4). Panels E1–E3 (right column) depict efficacy endpoints: graft survival free from failure (E1), rejection-free survival (biopsy-proven acute rejection) (E2), and *de novo* donor-specific antibody (dnDSA) development (E3). For each endpoint, unadjusted Kaplan–Meier curves are shown separately for fast and slow exposure phenotypes, with the number at risk displayed below each plot and log-rank P values provided within panels.

Supplementary Figure 4 Longitudinal estimated glomerular filtration rate (eGFR) trajectories according to tacrolimus exposure phenotype. Overall eGFR trajectories for the fast (blue) and slow (pink) dose-adjusted exposure phenotypes are shown over the follow-up period using locally smoothed curves. Time-weighted mean eGFR did not differ significantly between groups (P = 0.315).
