# Supplementary figures and images for: Impact of dose-adjusted tacrolimus exposure phenotype on outcomes in kidney transplantation: a large-scale multicenter cohort study
Source: Front Immunol. 2026 Apr 15;17:1811657. doi: 10.3389/fimmu.2026.1811657 (PMC13124564; doi:10.3389/fimmu.2026.1811657)

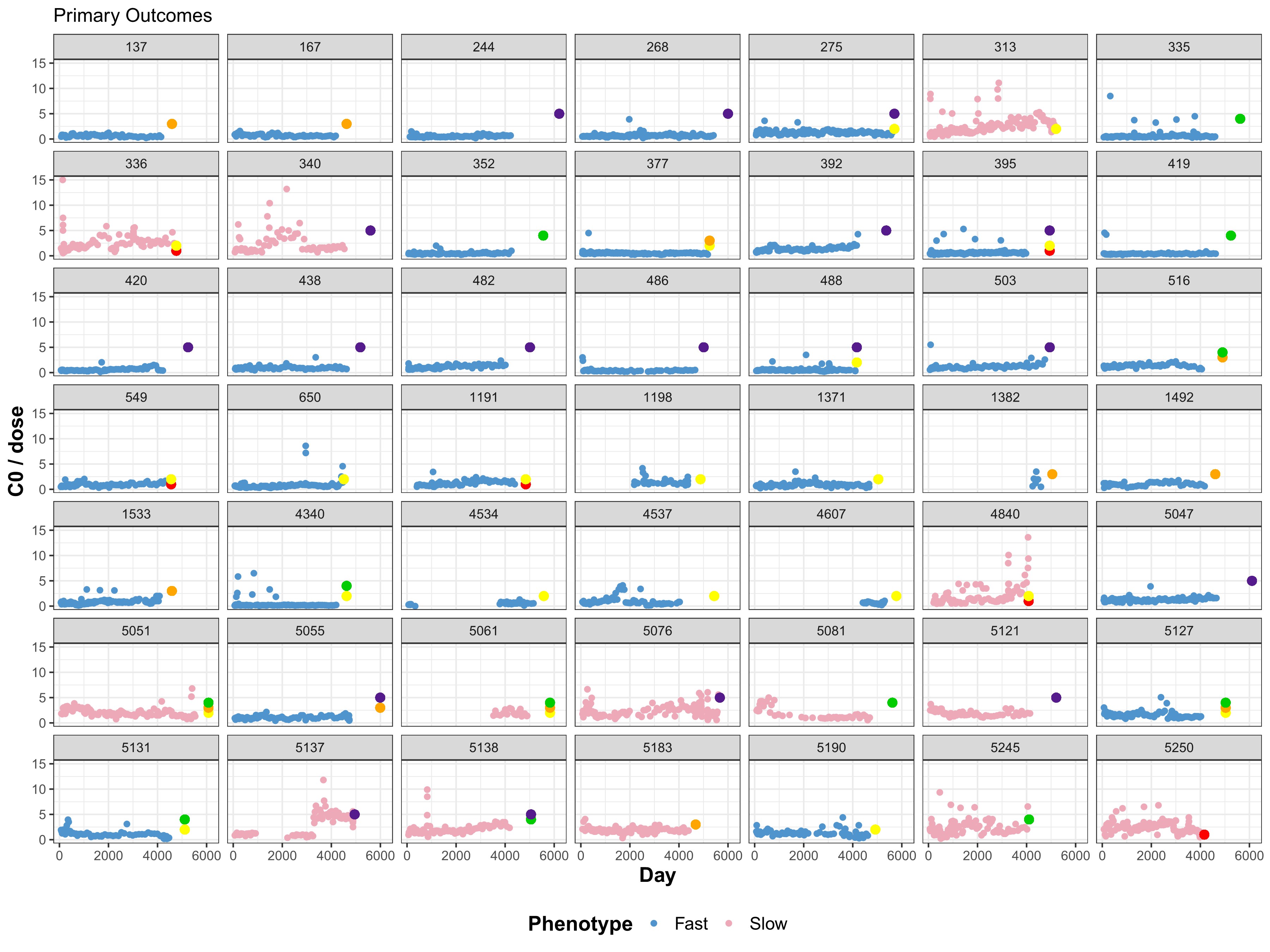

Supplement: Supplementary Figure 1 — Pre-event tacrolimus C0/dose trajectories among kidney transplant recipients with the longest follow-up. Serial tacrolimus C0/dose measurements from postoperative day 60 until just before the first occurrence of a clinical outcome or censoring are displayed for the 25 patients with the longest follow-up. Each line represents an individual trajectory, color-coded by outcome type: death (red), graft failure (yellow), biopsy-proven acute rejection (orange), de novo donor-specific antibody development (green), and severe infection requiring hospitalization (purple). Trajectories are overlaid on the fast and slow dose-adjusted exposure phenotype bands, and all patients remain within their originally assigned exposure phenotype without crossing between bands during the pre-event period, indicating no evidence of phenotype switching prior to outcome occurrence. [file Image1.jpeg]

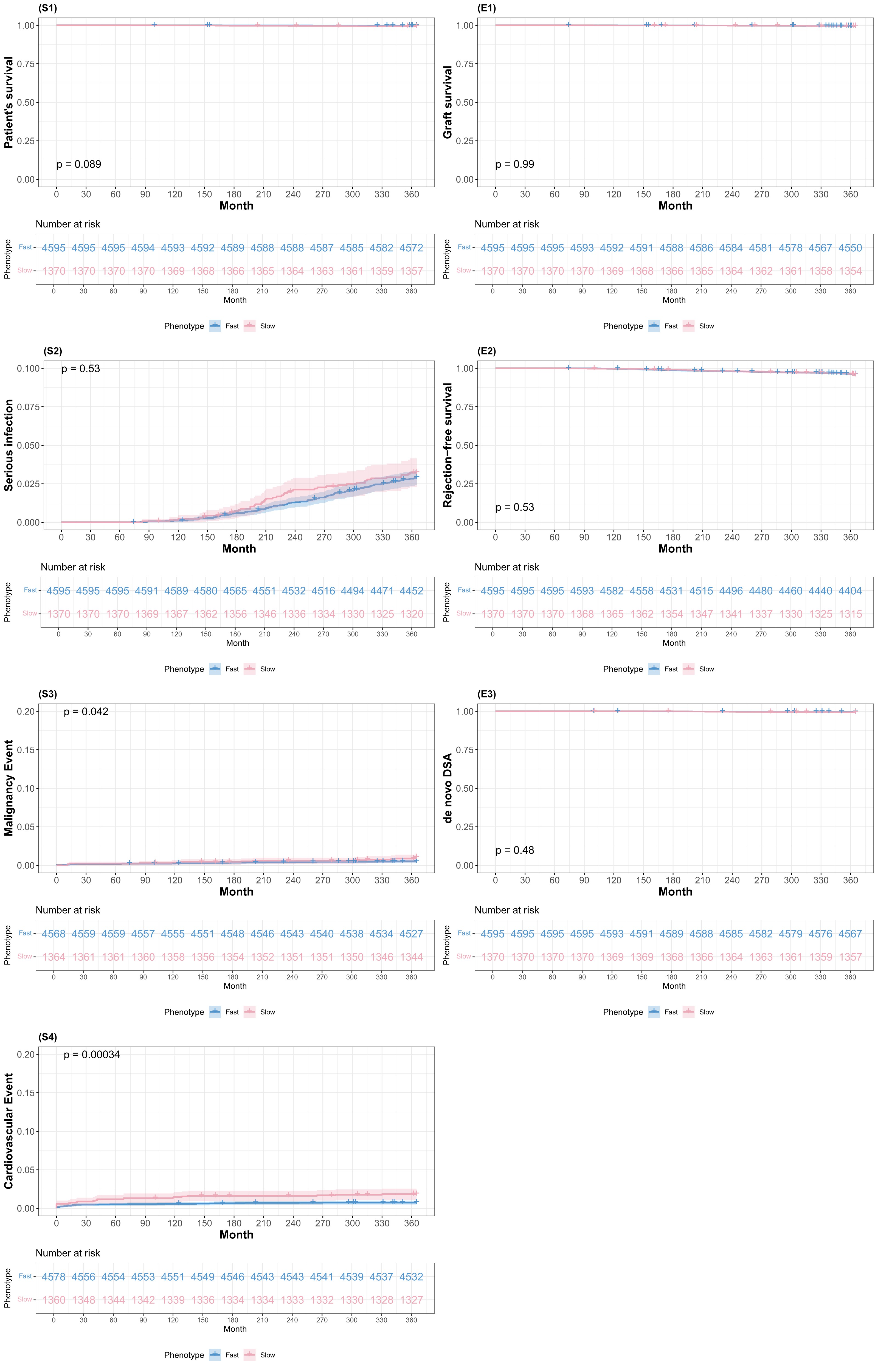

Supplement: Supplementary Figure 2 — Crude Kaplan-Meier estimates of the 1-year composite clinical endpoint comparing fast vs slow tacrolimus C0/dose trajectory-derived exposure phenotypes. Panels S1–S4 (left column) depict safety endpoints: patient survival (S1), serious infection requiring hospitalization (S2), malignancy (S3), and cardiovascular events (S4). Panels E1–E3 (right column) depict efficacy endpoints: graft survival free from failure (E1), rejection-free survival (biopsy-proven acute rejection) (E2), and de novo donor-specific antibody (dnDSA) development (E3). For each endpoint, unadjusted Kaplan–Meier curves are shown separately for fast and slow exposure phenotypes, with the number at risk displayed below each plot and log-rank P values provided within panels. [file Image2.jpeg]

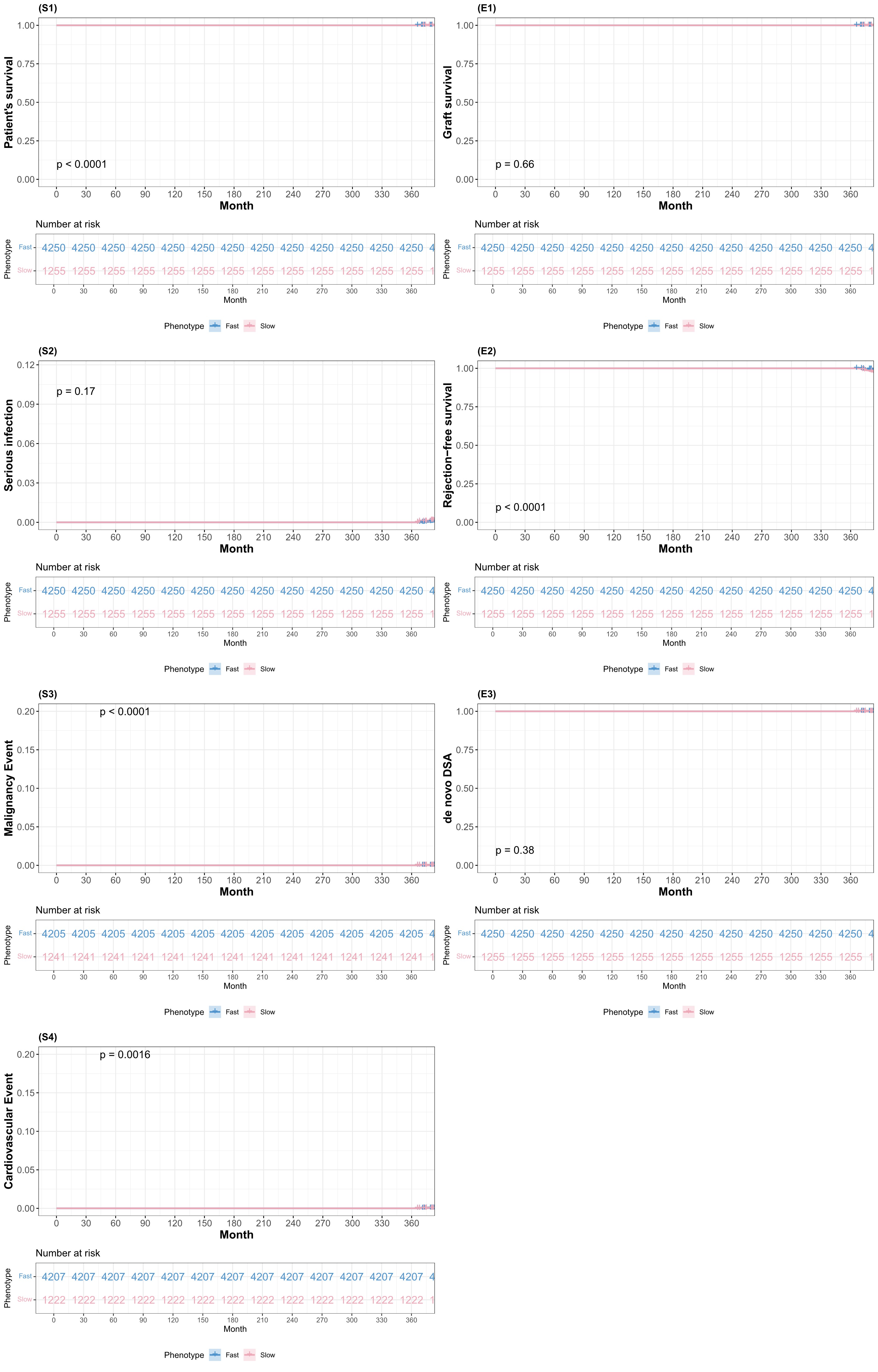

Supplement: Supplementary Figure 3 — Crude Kaplan-Meier estimates of the 2–6 year composite clinical endpoint comparing fast vs slow tacrolimus C0/dose trajectory-derived exposure phenotypes. Panels S1–S4 (left column) depict safety endpoints: patient survival (S1), serious infection requiring hospitalization (S2), malignancy (S3), and cardiovascular events (S4). Panels E1–E3 (right column) depict efficacy endpoints: graft survival free from failure (E1), rejection-free survival (biopsy-proven acute rejection) (E2), and de novo donor-specific antibody (dnDSA) development (E3). For each endpoint, unadjusted Kaplan–Meier curves are shown separately for fast and slow exposure phenotypes, with the number at risk displayed below each plot and log-rank P values provided within panels. [file Image3.jpeg]

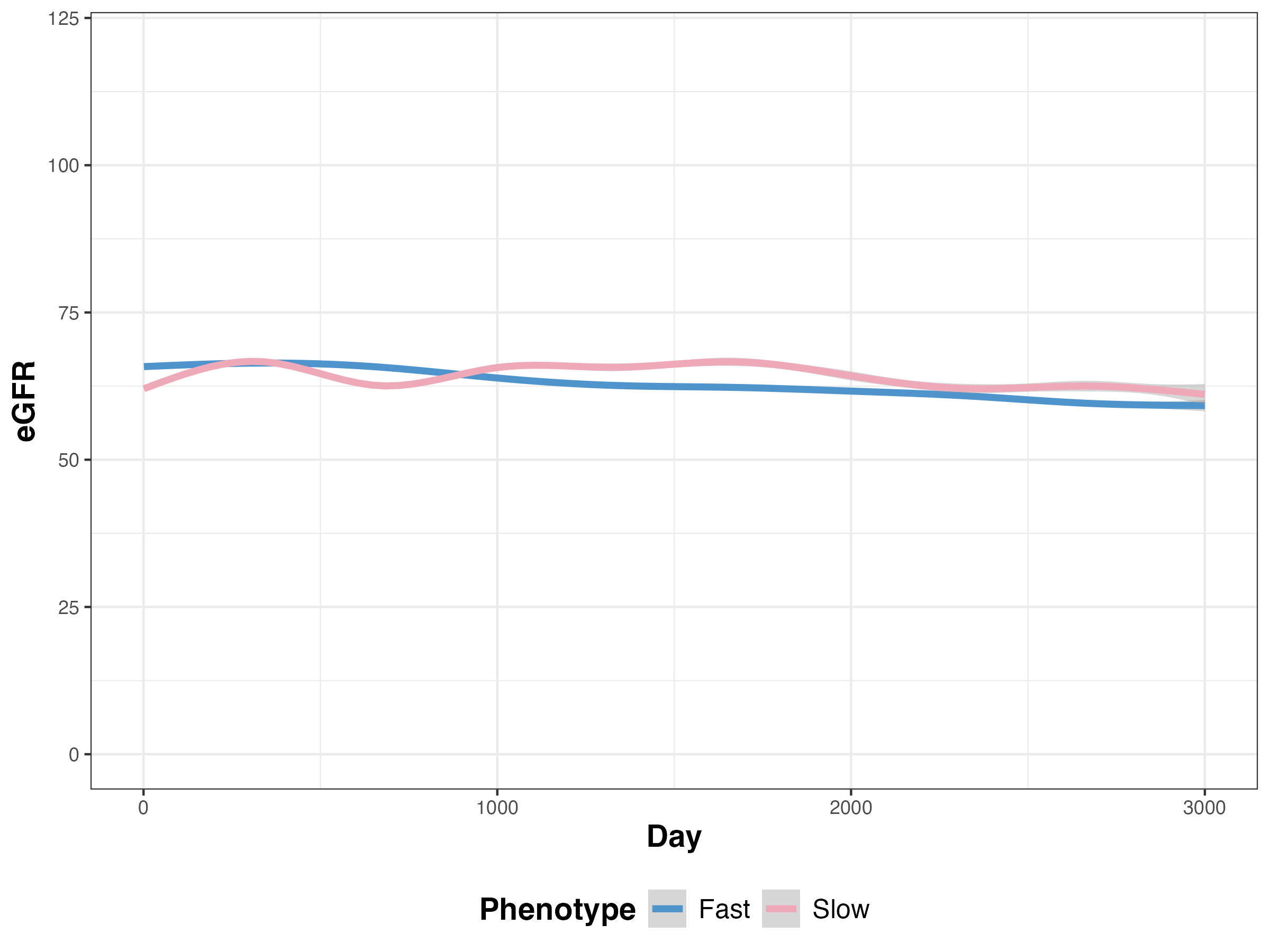

Supplement: Supplementary Figure 4 — Longitudinal estimated glomerular filtration rate (eGFR) trajectories according to tacrolimus exposure phenotype. Overall eGFR trajectories for the fast (blue) and slow (pink) dose-adjusted exposure phenotypes are shown over the follow-up period using locally smoothed curves. Time-weighted mean eGFR did not differ significantly between groups (P = 0.315). [file Image4.jpeg]
